# Real-world Evidence for Improvements in Inflammation and Anemia Biomarkers After the Initiation of GLP-1RA in Patients Receiving Hemodialysis

**DOI:** 10.1016/j.xkme.2026.101476

**Published:** 2026-07-17

**Authors:** Suman Lama, Sheetal Chaudhuri, Derek Blankenship, Andrea Nandorine Ban, Len Usvyat, Roberto Pecoits-Filho, Benjamin E. Hippen

**Affiliations:** 1 Renal Research Institute, New York, NY; 2 Pontifícia Universidade Católica do Paraná, School of Medicine, Curitiba, Brazil; 3 Fresenius Medical Care, Global Medical Office, Waltham, MA

**Keywords:** Glucagon-like peptide-1 receptor agonist, anemia, inflammatory markers, C-reactive protein, end-stage kidney failure, erythropoietin resistance

## Abstract

**Rationale & Objective:**

Glucagon-like peptide-1 receptor agonists (GLP-1RAs) are increasingly used for diabetes and obesity but have not been studied in patients with kidney failure with replacement therapy receiving hemodialysis in prospective trials. GLP-1RAs may influence inflammatory pathways relevant to anemia, although their effects in hemodialysis patients remain unclear.

**Study Design:**

Retrospective matched cohort study.

**Setting & Participants:**

Data were derived from a large US dialysis provider (Fresenius Kidney Care). We identified 2,468 adult patients who initiated GLP-1RA therapy between January 1, 2023, and November 1, 2024, and matched 1:1 to nonusers using propensity scores based on demographic characteristics, comorbidities, and baseline laboratory values.

**Exposure:**

Initiation of GLP-1RA therapy.

**Outcomes:**

Longitudinal changes over 12 months in inflammatory markers (neutrophil-to-lymphocyte ratio, white blood cell count, and albumin level) and anemia parameters (hemoglobin level, ferritin level, transferrin saturation value, erythropoiesis-stimulating agent (ESA) dose, and erythropoietin resistance index).

**Analytical Approach:**

Linear mixed models were used to compare longitudinal trajectories between matched groups.

**Results:**

Compared with matched controls, GLP-1RA users demonstrated reductions in systemic inflammation, characterized by lower neutrophil-to-lymphocyte ratio (3.97 vs 4.64, with a mean difference of −0.67 [95% CI, −0.89 to −0.45]) and white blood cell counts at 12 months, alongside greater improvements in serum albumin level (3.92 vs 3.86 g/dL, with a mean difference of 0.06 [95% CI, 0.04-0.08]). Although hemoglobin levels remained comparable between groups (10.9 vs 10.84 g/dL, with a mean difference of 0.06 [95% CI, -0.004 to 0.12]), GLP-1RA users had lower cumulative ESA exposure (1,881.9 vs 2,005.8 μg; mean difference of −123.9 μg, [95% CI, −201.7 to −46.1] μg) and a modestly reduced rate of erythropoietin resistance index compared with nonusers. Additionally, iron stores (ferritin level and transferrin saturation value) were consistently higher in the GLP-1RA group.

**Conclusions:**

In patients receiving maintenance hemodialysis, GLP-1RA agonist use was associated with reductions in inflammatory markers and lower ESA requirements; however, as an observational study, causality cannot be inferred. These findings suggest GLP-1RAs may have potential benefits in managing inflammation and inflammation-mediated anemia in kidney failure.

## Plain Language Summary

Patients receiving hemodialysis often experience inflammation and anemia, which can make treatment more difficult and affect outcomes. Glucagon-like peptide-1 receptor agonists (GLP-1RAs), commonly used for diabetes and weight management, may also have anti-inflammatory effects. We studied whether starting GLP-1RA therapy in patients on hemodialysis was associated with changes in markers of inflammation, nutrition, and anemia over 1 year, compared with similar patients not receiving these medications. We found that patients using GLP-1RAs showed consistent improvements in inflammatory markers and indicators of iron use, along with lower requirements for anemia medications, while maintaining similar hemoglobin levels. These findings suggest that GLP-1RAs may have benefits beyond glucose control, though further studies are needed to confirm their clinical impact.

## Introduction

Glucagon-like peptide-1 receptor agonists (GLP-1RAs) have emerged as valuable therapeutic options in managing type 2 diabetes and obesity because of their glucose-lowering, weight-reducing, and anti-inflammatory properties. Their use in patients with chronic kidney disease (CKD), including those with kidney failure with replacement therapy (KFRT) receiving maintenance hemodialysis (HD), is of growing clinical interest^1^ but remains limited and under-investigated compared with the general population of patients with obesity, diabetes, and heart failure without CKD. In CKD populations, GLP-1RA therapies are increasingly considered for glycemic control, cardiovascular risk reduction, and slowing CKD progression. The exact mechanisms of these effects are unclear, but the anti-inflammatory effects of GLP-1RA in general patient populations are now well established.^2^^,^^3^

Systemic inflammation is associated with poor outcomes in patients receiving HD and contributes to anemia, particularly because of ESA resistance and alterations in iron availability. The potential effect of GLP-1RAs on inflammation-related anemia parameters in this population has not been investigated.^4^ We conducted an exploratory analysis to evaluate whether initiation of GLP-1RA therapy in adults with KFRT receiving HD was associated with changes in anemia markers and anemia management over 12 months compared with matched nonusers.

## Methods

### Study Design and Population

This retrospective matched cohort study included 76,011 adult patients with KFRT who were treated at a large US dialysis provider (Fresenius Kidney Care, Waltham, United States) between January 1, 2023, and November 1, 2024. A cohort of patients with any active prescription after the initiation of dialysis (regardless of drug, dose, or indication) for any GLP-1RA was identified (n=2,468). GLP-1RA users were identified based on active prescriptions for agents including semaglutide, tirzepatide, dulaglutide, liraglutide, and exenatide. The cohort was predominantly composed of patients receiving newer-generation therapies: semaglutide (48.1%), dulaglutide (29.2%), and tirzepatide (14.3%). Older agents like liraglutide and exenatide accounted for the remaining minority (8.4%) of prescriptions. The duration of pre-KFRT therapy with GLP-1RA was not available. The index date for GLP-1RA users was defined as the first recorded administration of a GLP-1RA during the study period.

A comparator cohort of non-GLP-1RA users was selected from the same HD population. For these patients, the index date was assigned, so the distribution of index dates matched that of the GLP-1RA cohort. This approach ensured that comparator patients had a similar time frame of observation relative to their assigned index date as GLP-1RA users had relative to treatment initiation. To evaluate the effects of sustained therapy, we restricted our analysis to a per-protocol cohort, requiring patients to have received uninterrupted in-center HD for 12 months following the index date and remain on GLP-1RA medication for the entire 12 months (365 days). Individuals were excluded if they had incomplete follow-up during this period, changed modality, discontinued the medications, died, or underwent kidney transplantation.

This project was reviewed and approved by the WCG Review Board (Cary; Work Order# 1-1899937-1). It was determined by the independent review board that this analysis was exempt because of deidentification of data, and consent was not required per Title 45 Code of Federal Regulations part 46.104(d)(4) in the United States. The analysis adhered to the Declaration of Helsinki.

### Statistical Analysis

Baseline patient characteristics (Table 1) were described using means and standard deviations for continuous variables and percentages for categorical variables. The primary analysis was conducted using the propensity score-matched cohort. For each outcome and time point, means and standard deviations (SDs) were calculated for GLP-1RA users and matched nonusers.

**Table 1:** Baseline Characteristics of the Population of Patients Receiving HD and Propensity Score Matched Patients

Parameter	Patient population			PSM matched		
	Mean ± SD or n (%)		Standardized mean difference	Mean ± SD or %		Standardized mean difference
	Non-GLP1 user	GLP-1RA		Non-GLP1 user	GLP-1RA	
Patient, n (%)	73,328 (96.5%)	2,683 (3.5%)		2,468	2,468	
Demography						
Age (y)	63.9 ± 13.9	60.8 ± 12.1	−0.161	61.2 ± 13.1	61.3 ± 11.9	−0.012
Dialysis vintage (y)^a^	4.9 ± 4.4	2.5± 3.2	−0.064	2.4 ± 2.5	2.5 ± 3.3	−0.016
Female	30,769 (42.0%)	1,261 (47.0%)	−0.001	1,150 (46.6%)	1,148 (46.5%)	−0.002
White race	37,046 (50.5%)	1,379 (51.4%)	−0.004	1,289 (52.2%)	1,279 (51.8%)	−0.008
Black race	28,352 (38.7%)	922 (34.4%)	0.010	815 (33.0%)	840 (34.0%)	0.021
Other race	6,159 (8.4%)	274 (10.2%)	−0.005	265 (10.7%)	253 (10.3%)	−0.017
Unknown race	1771 (2.4%)	108 (4.0%)	−0.001	99 (4.0%)	96 (3.9%)	−0.007
BMI (kg/m^2^)	28.4 ± 7.1	35.4 ± 8.6	0.700	35.3 ± 10.1	34.8 ± 8.2	0.089
Laboratory parameters						
TSAT value (%)	33.56 ± 15.68	29.6 ± 14.79	−0.291	29.56 ± 13.28	29.81 ± 14.89	−0.019
Albumin level (g/dL)	3.92 ± 0.35	3.85 ± 0.37	−0.015	3.84 ± 0.39	3.85 ± 0.38	−0.043
NLR value	4.09 ± 3.15	4.38 ± 3.12	0.205	4.43 ± 2.87	4.4 ± 3.17	0.065
Hemoglobin level (g/dL)	10.75 ± 1.27	10.47 ± 1.4	0.008	10.48 ± 1.39	10.47 ± 1.4	0.006
Iron level (μg/dL)	75.3 ± 34.92	69.27 ± 31.93	−0.536	69.2 ± 29.39	69.65 ± 32.15	−0.016
Ferritin level (ng/mL)	906.53 ± 488.82	689.07 ± 468.2	−3.569	697.06 ± 446.24	700.14 ± 467.89	−0.007
WBC count (1,000/μL)	6.7 ± 2.45	7.65 ± 2.48	0.139	7.73 ± 3.64	7.61 ± 2.47	0.056
Medication						
ESA dose (μg)	27.0 ± 30.6	16.6± 25.6	0.232	17.0 ± 22.6	17.2 ± 26.1	0.008
IV Iron (mg)	54.3 ± 37.4	44.4 ± 41.5	−0.750	45.3 ± 38.5	45.6 ± 41.6	−0.019
Insurance						
Commercial Insurance	5,647 (7.7%)	349 (13.0%)	−0.001	309 (12.5%)	306 (12.4%)	−0.004
Medicaid insurance	7,197 (9.8%)	278 (10.4%)	0.005	244 (9.9%)	256 (10.4%)	0.016
Medicare insurance	57,707 (78.7%)	1,962 (73.1%)	0.002	1,813 (73.5%)	1,817 (73.6%)	0.004
Others^b^	2,777 (3.8%)	94 (3.5%)	0.016	102 (4.1%)	89 (3.6%)	0.026
Comorbidities						
Diabetes	46,239 (63.1%)	2,172 (81.0%)	−0.031	2,112 (85.6%)	2,036 (82.5%)	−0.070
Coronary artery disease	18,955 (25.8%)	633 (23.6%)	0.000	603 (24.4%)	604 (24.5%)	0.001
Peripheral artery disease	5,882 (8.0%)	109 (4.1%)	0.008	87 (3.5%)	106 (4.3%)	0.032
Congestive heart failure	18,555 (25.3%)	704 (26.2%)	−0.005	672 (27.2%)	660 (26.7%)	−0.011
Cerebrovascular disease	5,482 (7.5%)	124 (4.6%)	0.008	103 (4.2%)	123 (5.0%)	0.034
Myocardial infarction	12,699 (17.3%)	502 (18.7%)	−0.004	491 (19.9%)	480 (19.4%)	−0.012
History of malignancy	3,014 (4.1%)	83 (3.1%)	−0.001	83 (3.4%)	81 (3.3%)	−0.004
ERI	2.7 ± 1.8	2.3 ± 1.5	0.000	2.3 ± 1.6	2.3 ± 1.5	0.000

*Note:* Data are presented as mean ± standard deviation (SD) for continuous variables and frequency (%) for categorical variables. The standardized mean difference (SMD) compares the matched GLP-1 receptor agonist (GLP-1RA) and nonuser groups; an absolute SMD < 0.1 indicates well-balanced groups.

Abbreviations: BMI, body mass index; ERI, erythropoietin resistance index; GLP-1RA, glucagon-like peptide-1 receptor agonists; HD, hemodialysis; IV, intravenous; NLR, neutrophil-to-lymphocyte ratio; PSM, propensity score matching; TSAT, transferrin saturation; WBC, white blood cell.

a Dialysis vintage was defined as the time from first dialysis treatment to the GLP-1RA initiation (index) date.

b Others' insurance types included insurance like veterans affairs, self-pay, and exchange plans, etc.

#### Propensity Score Matching

To minimize the effects of selection bias and potential confounding, we performed propensity score matching (PSM) to balance the baseline characteristics between the treatment and control groups. Missing data in baseline covariates were handled using multiple imputations by chained equations. Only baseline covariates were imputed; outcomes were not imputed. A multivariable logistic regression model was then developed to estimate the propensity score, defined as the probability of treatment assignment, for each patient within each imputed dataset. The model included the following covariates: demographic characteristics (age, gender, race), dialysis vintage, insurance plan type, body mass index (BMI, kg/m^2^), mean serum albumin level (g/dL), mean white blood cell (WBC count, ×10^3^/μL), mean hemoglobin level (g/dL), mean serum iron level (μg/dL), mean transferrin saturation value (%), mean serum ferritin level (ng/mL), neutrophil-to-lymphocyte ratio (NLR), erythropoiesis-stimulating agent (ESA), erythropoietin resistance index (ERI),^5^ relevant medication dosages (mean ESA dose [μg], mean serum iron level [μg/dL]), and a history of major comorbidities that were active during our study period. Insurance status was categorized into 4 primary, mutually exclusive groups: Medicare, Medicaid, Commercial, and Others. For the purpose of baseline characterization, patients with “Other” insurance types included insurance like Veterans Affairs, self-pay, Exchange plan, etc.

To calculate the ERI value, the total monthly ESA dosage was first divided by the number of weeks in that month to normalize it to a weekly level. The mean weekly ESA value for each period was then computed and subsequently divided by the patient’s mean body weight and by the average hemoglobin level. All ESA doses were converted to methoxy polyethylene glycol-epoetin beta (Mircera) dosing (darbepoetin: μg/1.28; epoetin alfa: μg/244.51).

The logit of the propensity scores was averaged across the imputed datasets for each patient. Subsequently, we performed a 1:1 nearest-neighbor greedy matching on the averaged logit propensity score using a caliper of 0.1 and an exact match on the calendar quarter to create a balanced cohort. The adequacy of the matching was confirmed by the match rate and by assessing the balance of all covariates before and after the matching process with standardized differences (Table 1).

#### Longitudinal Analysis

Following propensity score matching, we used linear mixed models to evaluate the longitudinal changes in outcomes between the treatment and control groups over the 365-day follow-up period. To accommodate diverse, nonlinear biological trajectories without overfitting and ensure comparability across all outcomes, time was modeled categorically (baseline, 30, 60, 90, 180, 270, and 365 days). Accordingly, models included fixed effects for treatment, time, and their interaction. To account for the correlation of repeated measurements within each patient and match, a corresponding subject- and match-specific random intercept was included. This analysis was performed for multiple outcomes, including hemoglobin level, ERI value, albumin level, and others. Post hoc analyses of least-squares means with a Bonferroni multiple testing correction were used to assess differences between the groups at each time point.

#### Outcomes

The primary outcomes were changes in serum albumin level (g/dL), WBC count (×10^3^/μL), NLR value, mean ESA dose (μg), cumulative ESA dose (μg), ERI value, iron level (μg/dL), hemoglobin level (g/dL), transferrin saturation %, ferritin (ng/mL), and intravenous (IV) iron (mg) over the 12-month follow-up. Laboratory and medication data were extracted at 1, 3, 6, 9, and 12 months after the index date.

#### Sensitivity Analyses

As sensitivity analyses, we incorporated time-varying covariates into the linear mixed-effects models to account for potential time-dependent confounding. Specifically, body weight measured at each follow-up time point was included as a time-varying covariate to account for potential GLP-1RA-associated weight change. Additionally, for anemia-related outcomes, IV iron dose at each time point was included as a time-varying covariate to account for differences in iron supplementation over time. These models were otherwise specified identically to the primary analyses.

#### Software

SAS Enterprise Guide 7.1 (SAS Institute Inc) was used to perform propensity score matching and linear mixed model analysis. Multiple imputation was done using a SAS procedure. Propensity score matching was done using PROC PS MATCH; PROC MIXED was used to run a linear mixed model. Python version 3.10.14 (Python Software Foundation, Delaware, United States) was used for data manipulation and visualization.

## Results

### Cohort and Follow-up

From an initial population of 76,011 eligible patients receiving HD, 2,683 (3.5%) GLP-1RA initiators were identified. Of these, 2,468 were successfully propensity score-matched 1:1 to 2,468 nonusers selected from the remaining patient cohort. The remaining 215 GLP-1RA initiators (8.0%) remained unmatched because of differences in baseline characteristics that could not be propensity matched in the control population. As detailed in Table S1, these unmatched patients were clinical outliers characterized by significantly higher BMI (42.4 vs 28.4 kg/m^2^), younger age, and shorter dialysis vintage compared with the available control pool. Baseline characteristics were well balanced after matching (Table 1). Notably, the use of other glucose-lowering therapies was well-balanced between the groups; for example, baseline dipeptidyl peptidase-4 inhibitor use was 11.1% in the GLP-1RA cohort compared with 9.8% in the matched non-user group, suggesting broadly comparable use of alternative glucose-lowering therapies between cohorts. The GLP-1RA cohort primarily utilized injectable semaglutide (Ozempic), reflecting current clinical prescribing patterns in the KFRT population.

### Inflammatory and Nutritional Biomarkers

**Figure 1:**
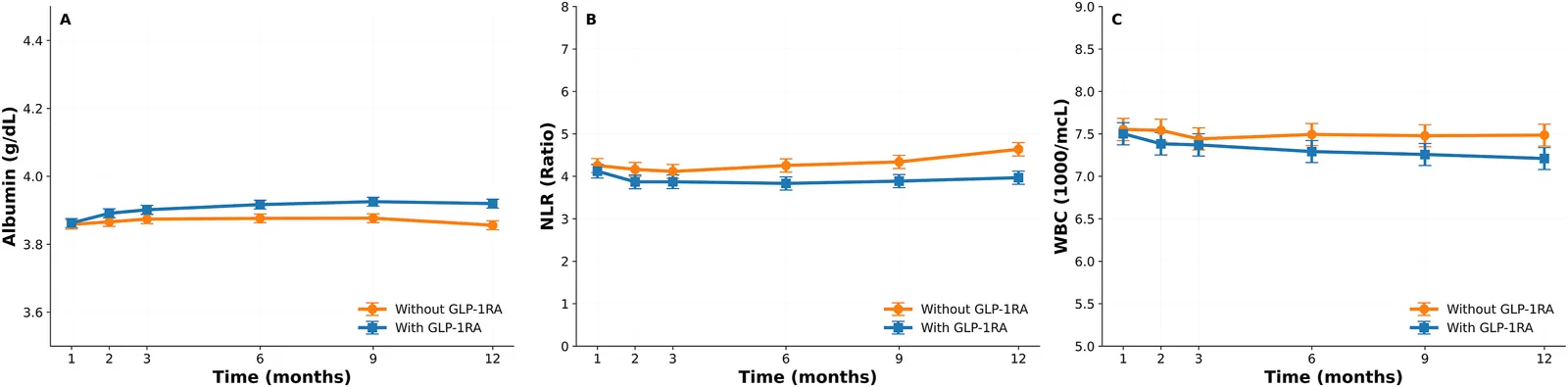
Longitudinal trends in inflammatory and nutritional biomarkers among glucagon-like peptide-1 receptor agonist (GLP-1RA) users and matched controls over 12 months. (A) Albumin, (B) neutrophil-to-lymphocyte ratio (NLR), and (C) white blood cell (WBC) count. Points represent mean values and error bars indicate 95% confidence intervals.

Longitudinal analysis demonstrated consistent directional improvements in markers of inflammation and nutrition among patients treated with GLP-1RAs compared with matched controls (Fig 1, Table 2). NLR, a marker of systemic inflammation, decreased in the GLP-1RA group compared with matched controls through month 12 (3.97 vs 4.64), with a mean difference of −0.67 (95% confidence interval [CI], −0.89 to −0.45) (Fig 1B, Table 2). Similarly, WBC counts declined in the GLP-1RA group compared with matched controls from month 6 through month 12 (7.21 vs 7.49 × 10^3^/μL; mean difference −0.28, 95% CI, −0.46 to −0.10) (Fig 1C, Table 2). The serum albumin level rose from 3.85 g/dL at baseline to 3.92 g/dL at 12 months in the GLP-1RA group and remained consistently higher than the control group with a mean difference of 0.06 (95% CI, 0.04-0.08) (Fig 1A, Table 2).

**Table 2:** Longitudinal Trends in Inflammatory and Nutritional Biomarker Status Over 12 Months

Outcome	Time	Treatment mean (95% CI)	Control mean (95% CI)	Mean difference (95% CI)
Albumin level (g/dL)	Baseline	3.85 (3.83-3.86)	3.83 (3.82-3.85)	0.02 (0.001-0.04)
	1 mo	3.86 (3.85-3.88)	3.86 (3.84-3.87)	0.00 (−0.02 to 0.02)
	2 mo	3.89 (3.88-3.90)	3.87 (3.85-3.88)	0.02 (0.001-0.04)
	3 mo	3.90 (3.89-3.91)	3.87 (3.86-3.89)	0.03 (0.01-0.05)
	6 mo	3.92 (3.90-3.93)	3.88 (3.86-3.89)	0.04 (0.02-0.06)
	9 mo	3.93 (3.91-3.94)	3.88 (3.86-3.89)	0.05 (0.03-0.07)
	12 mo	3.92 (3.91-3.93)	3.86 (3.84-3.87)	0.06 (0.04-0.08)
NLR value (ratio)	Baseline	4.39 (4.24-4.53)	4.59 (4.45-4.74)	−0.2 (−0.4 to 0.004)
	1 mo	4.12 (3.96-4.28)	4.25 (4.09-4.42)	−0.13 (−0.36 to 0.10)
	2 mo	3.87 (3.71-4.03)	4.16 (4.00-4.33)	−0.29 (−0.52 to −0.06)
	3 mo	3.87 (3.71-4.03)	4.12 (3.95-4.28)	−0.25 (−0.48 to −0.02)
	6 mo	3.83 (3.68-3.99)	4.26 (4.10-4.41)	−0.43 (−0.65 to −0.21)
	9 mo	3.89 (3.73-4.04)	4.34 (4.18-4.50)	−0.45 (−0.67 to −0.23)
	12 mo	3.97 (3.81-4.12)	4.64 (4.48-4.79)	−0.67 (−0.89 to −0.45)
WBC count (1,000/μL)	Baseline	7.62 (7.49-7.75)	7.76 (7.63-7.89)	−0.14 (−0.32 to 0.04)
	1 mo	7.50 (7.37-7.63)	7.55 (7.42-7.68)	−0.05 (−0.24 to 0.14)
	2 mo	7.38 (7.25-7.51)	7.54 (7.41-7.67)	−0.16 (−0.35 to 0.03)
	3 mo	7.37 (7.24-7.50)	7.44 (7.31-7.57)	−0.07 (−0.26 to 0.12)
	6 mo	7.29 (7.16-7.42)	7.49 (7.36-7.62)	−0.2 (−0.38 to −0.02)
	9 mo	7.26 (7.13-7.39)	7.48 (7.35-7.61)	−0.22 (−0.4 to −0.04)
	12 mo	7.21 (7.08-7.34)	7.49 (7.36-7.62)	−0.28 (−0.46 to −0.10)

Abbreviations: CI, confidence interval; NLR, neutrophil-to-lymphocyte ratio; WBC, white blood cell.

### Anemia Management and Iron Metabolism

**Figure 2:**
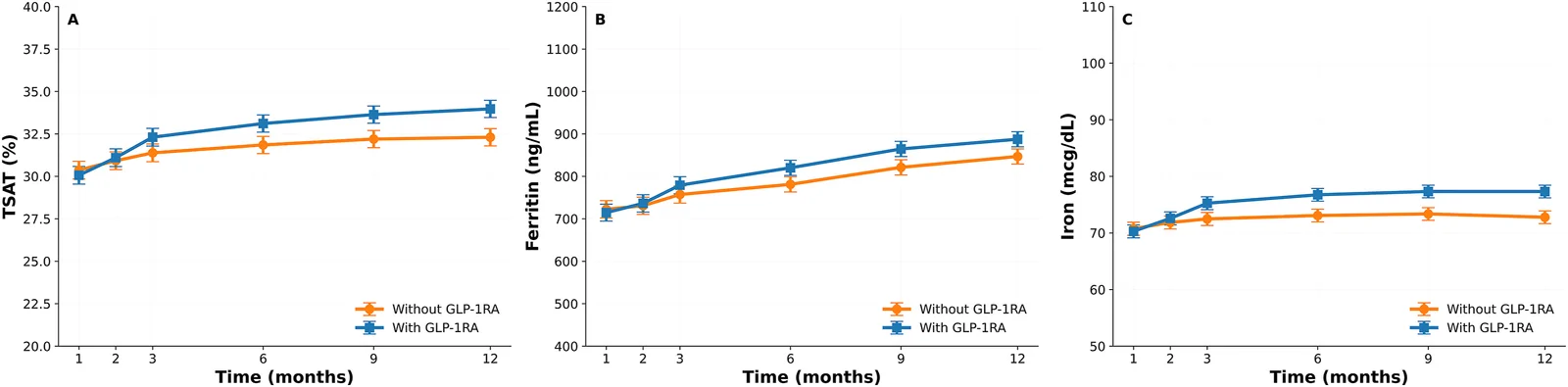
Longitudinal trends in iron metabolism parameters among glucagon-like peptide-1 receptor agonist (GLP-1RA) users and matched controls over 12 months. (A) Transferrin saturation (TSAT), (B) ferritin, and (C) serum iron. Points represent mean values, and error bars indicate 95% confidence intervals.

**Figure 3:**
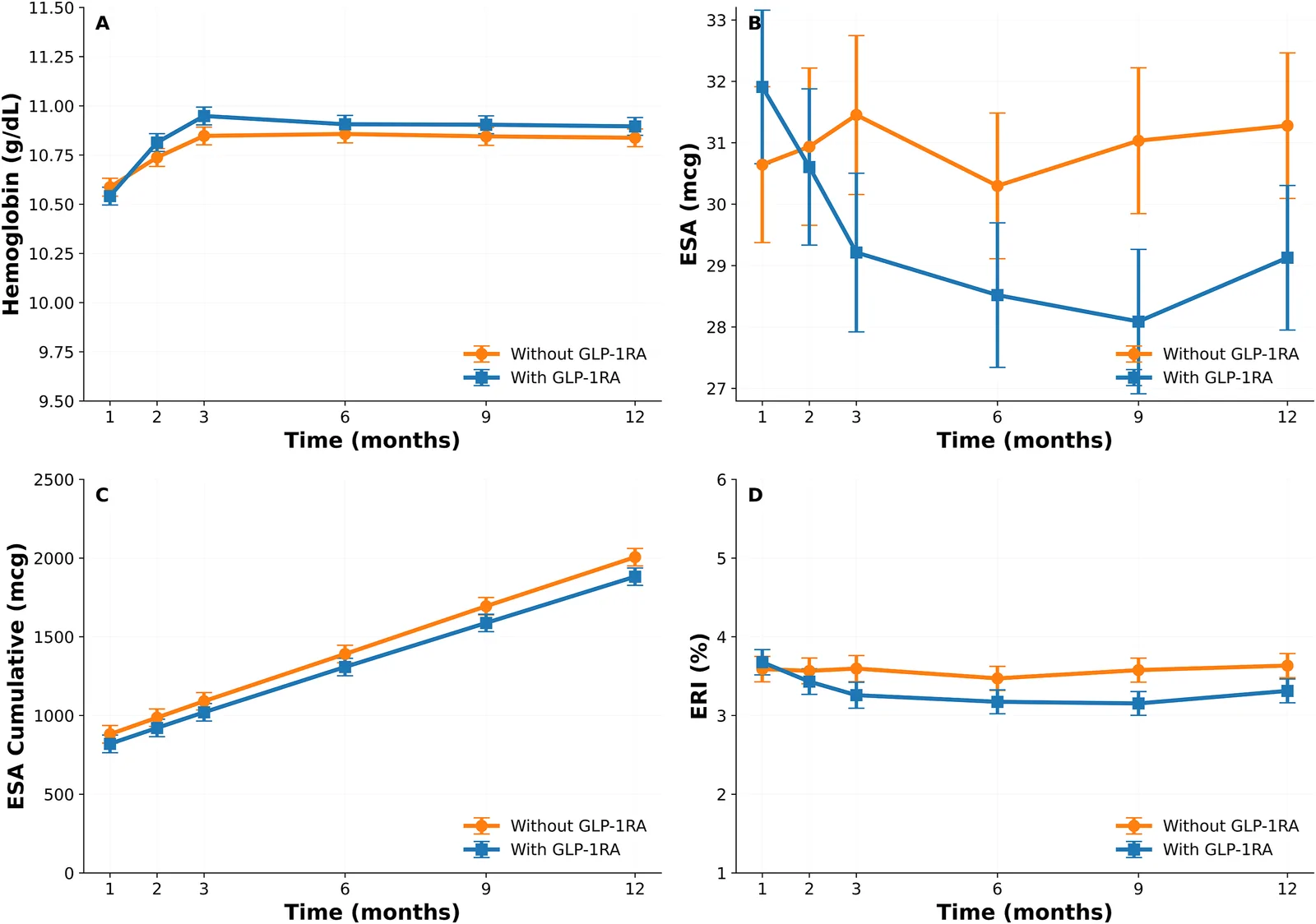
Longitudinal trends in anemia management and erythropoietic response among glucagon-like peptide-1 receptor agonist (GLP-1RA) users and matched controls over 12 months. (A) Hemoglobin levels, (B) erythropoiesis-stimulating agent (ESA) dose, (C) cumulative ESA exposure, and (D) erythropoietin resistance index (ERI). Points represent mean values and error bars indicate 95% confidence intervals.

**Figure 4:**
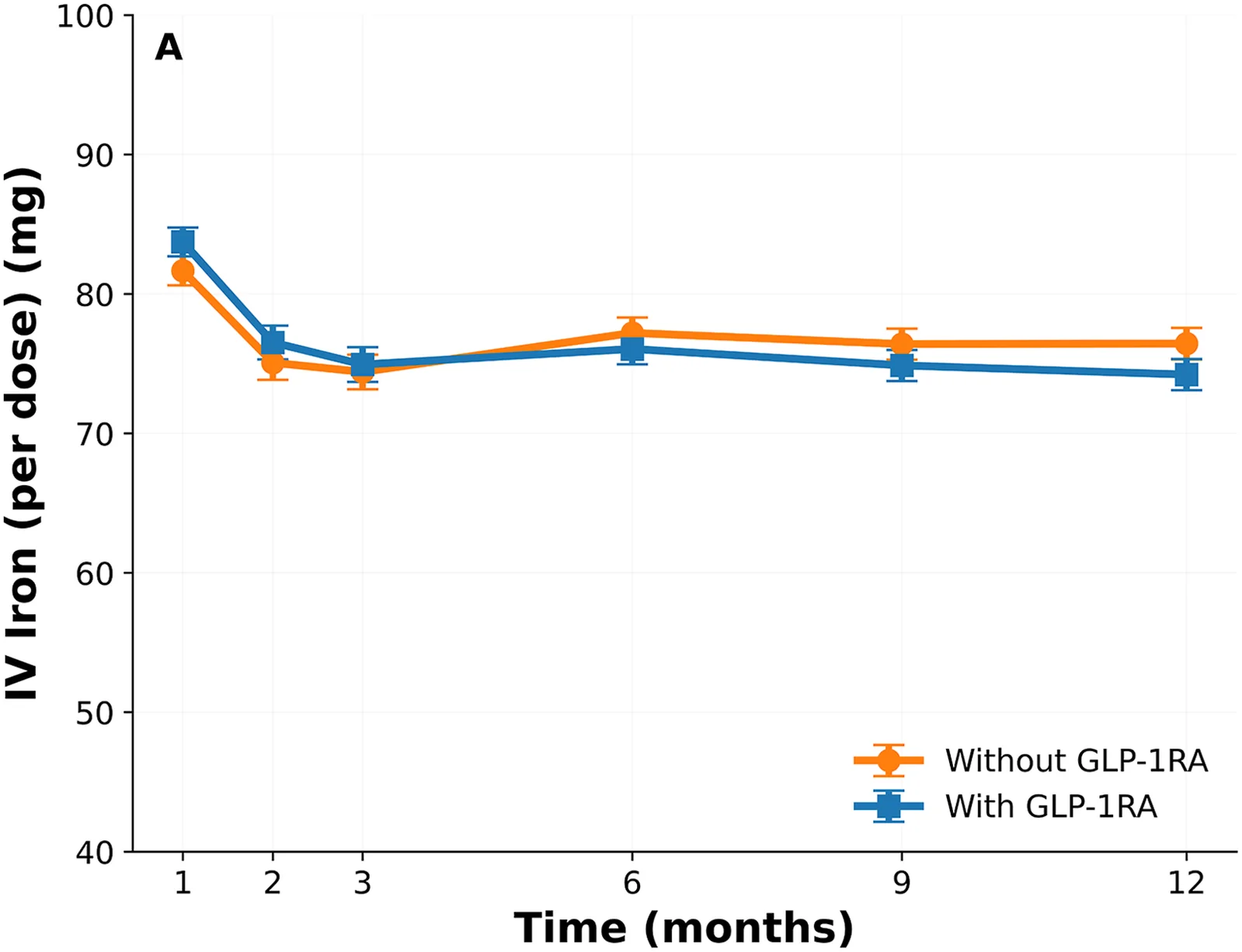
Longitudinal trends in intravenous (IV) iron dose among glucagon-like peptide-1 receptor agonist (GLP-1RA) users and matched controls over 12 months. Points represent adjusted mean values, and error bars indicate 95% confidence intervals.

The GLP-1RA group demonstrated improved erythropoietic efficiency and more favorable iron trajectories compared with matched controls (Figure 2, Figure 3, Figure 4, Table 3).

**Table 3:** Longitudinal Trends in Anemia Management and Iron Metabolism Over 12 Months

Outcome	Time	Treatment mean (95% CI)	Control mean (95% CI)	Mean difference (95% CI)
Hemoglobin level (g/dL)	Baseline	10.47 (10.42-10.51)	10.48 (10.43-10.52)	−0.01 (−0.07 to 0.05)
	1 mo	10.54 (10.50-10.59)	10.59 (10.54-10.63)	−0.05 (−0.11 to 0.01)
	2 mo	10.81 (10.77-10.86)	10.74 (10.69-10.78)	0.07 (0.006-0.13)
	3 mo	10.95 (10.90-10.99)	10.85 (10.80-10.89)	0.1 (0.04-0.16)
	6 mo	10.91 (10.86-10.95)	10.86 (10.81-10.90)	0.05 (−0.01 to 0.11)
	9 mo	10.90 (10.86-10.95)	10.84 (10.80-10.89)	0.06 (−0.004 to 0.12)
	12 mo	10.90 (10.85-10.94)	10.84 (10.79-10.88)	0.06 (−0.004 to 0.12)
ESA dose (μg)	Baseline	17.20 (16.10-18.30)	17.40 (16.30-18.50)	−0.20 (−1.76 to 1.36)
	1 mo	31.90 (30.70-33.20)	30.60 (29.40-31.90)	1.30 (−0.48 to 3.08)
	2 mo	30.60 (29.30-31.90)	30.90 (29.70-32.20)	−0.30 (−2.11 to 1.51)
	3 mo	29.20 (27.90-30.50)	31.50 (30.20-32.70)	−2.30 (−4.13 to −0.47)
	6 mo	28.50 (27.30-29.70)	30.30 (29.10-31.50)	−1.80 (−3.47 to −0.13)
	9 mo	28.10 (26.90-29.30)	31.00 (29.80-32.20)	−2.90 (−4.57 to −1.23)
	12 mo	29.10 (27.90-30.30)	31.30 (30.10-32.50)	−2.20 (−3.87 to −0.53)
Iron level (μg/dL)	Baseline	69.70 (68.60-70.80)	69.20 (68.10-70.30)	0.50 (−1.08 to 2.08)
	1 mo	70.30 (69.10-71.40)	70.70 (69.60-71.90)	−0.40 (−2.02 to 1.22)
	2 mo	72.60 (71.40-73.70)	71.90 (70.70-73.00)	0.70 (−0.92 to 2.32)
	3 mo	75.30 (74.10-76.40)	72.50 (71.30-73.60)	2.80 (1.18 to 4.42)
	6 mo	76.70 (75.60-77.90)	73.10 (72.00-74.20)	3.60 (2.02 to 5.18)
	9 mo	77.30 (76.20-78.40)	73.40 (72.20-74.50)	3.90 (2.32 to 5.48)
	12 mo	77.30 (76.20-78.40)	72.80 (71.70-73.90)	4.50 (2.92-6.08)
IV iron (per dose) (mg)	Baseline	45.90 (44.90-46.90)	45.10 (44.10-46.10)	0.80 (−0.61 to 2.21)
	1 mo	83.70 (82.70-84.80)	81.70 (80.60-82.70)	2.00 (0.54-3.46)
	2 mo	76.50 (75.30-77.70)	75.10 (73.80-76.30)	1.40 (−0.32 to 3.12)
	3 mo	74.90 (73.70-76.20)	74.40 (73.20-75.60)	0.50 (−1.25 to 2.25)
	6 mo	76.00 (74.90-77.20)	77.20 (76.10-78.30)	−1.20 (−2.76 to 0.36)
	9 mo	74.90 (73.80-76.00)	76.40 (75.30-77.50)	−1.50 (−3.07 to 0.07)
	12 mo	74.20 (73.10-75.30)	76.40 (75.30-77.60)	−2.20 (−3.79 to −0.61)
TSAT value (%)	Baseline	29.90 (29.40-30.40)	29.60 (29.10-30.10)	0.3 (−0.42 to 1.02)
	1 mo	30.10 (29.50-30.60)	30.40 (29.80-30.90)	−0.3 (−1.04 to 0.44)
	2 mo	31.10 (30.60-31.60)	30.90 (30.40-31.40)	0.2 (−0.54 to 0.94)
	3 mo	32.30 (31.80-32.80)	31.40 (30.90-31.90)	0.9 (0.16-1.64)
	6 mo	33.10 (32.60-33.60)	31.90 (31.30-32.40)	1.2 (0.48-1.92)
	9 mo	33.60 (33.10-34.10)	32.20 (31.70-32.70)	1.4 (0.68-2.12)
	12 mo	34.00 (33.50-34.50)	32.30 (31.80-32.80)	1.7 (0.98-2.42)
ERI value (%)	Baseline	2.68 (2.52-2.85)	2.61 (2.45-2.78)	0.07 (−0.17 to 0.31)
	1 mo	3.68 (3.52-3.84)	3.59 (3.43-3.75)	0.09 (−0.14 to 0.32)
	2 mo	3.43 (3.27-3.59)	3.57 (3.40-3.73)	−0.14 (−0.37 to 0.09)
	3 mo	3.26 (3.09-3.42)	3.60 (3.43-3.76)	−0.34 (−0.57 to −0.11)
	6 mo	3.17 (3.02-3.33)	3.47 (3.32-3.62)	−0.30 (−0.52 to −0.08)
	9 mo	3.15 (3.00-3.30)	3.58 (3.42-3.73)	−0.43 (−0.65 to −0.21)
	12 mo	3.31 (3.16-3.46)	3.63 (3.48-3.79)	−0.32 (−0.54 to −0.10)
Ferritin level (ng/mL)	Baseline	700.90 (683.10-718.70)	697.40 (679.50-715.20)	3.50 (−21.70 to 28.70)
	1 mo	714.60 (694.60-734.60)	722.50 (702.10-742.90)	−7.90 (−36.40 to 20.60)
	2 mo	736.40 (716.00-756.90)	730.80 (710.50-751.10)	5.60 (−23.20 to 34.40)
	3 mo	779.10 (759.00-799.40)	757.30 (737.00-777.50)	21.80 (−6.79 to 50.4)
	6 mo	820.20 (802.30-838.10)	781.20 (763.30-799.10)	39.00 (13.70-64.30)
	9 mo	864.60 (846.70-882.50)	821.20 (803.30-839.10)	43.40 (18.10-68.70)
	12 mo	887.40 (869.60-905.30)	846.90 (829.00-864.80)	40.50 (15.2-65.8)
ESA cumulative dose (μg)	Baseline	N/A	N/A	N/A
	1 mo	820.10 (764.00-876.20)	881.00 (825.10-936.80)	−60.90 (−140.10 to 18.30)
	2 mo	921.00 (865.40-976.70)	986.30 (930.70-1,042.00)	−65.30 (−144.00 to 13.40)
	3 mo	1,020.50 (965.00-1,076.00)	1,090.60 (1,035.10-1,146.20)	−70.10 (−148.60 to 8.42)
	6 mo	1,308.20 (1,252.90-1,363.50)	1,390.60 (1,335.20-1,445.90)	−82.40 (−160.60 to −4.16)
	9 mo	1,587.90 (1,532.70-1,643.10)	1,694.30 (1,639.10-1,749.60)	−106.40 (−184.50 to −28.30)
	12 mo	1,882.00 (1,827.00-1,937.00)	2,005.80 (1,950.70-2,060.90)	−123.80 (−201.70 to −45.90)

Abbreviations: CI, confidence interval; ERI, erythropoietin resistance index; ESA, erythropoiesis-stimulating agent; IV, intravenous; N/A, not applicable; TSAT, transferrin saturation.

By month 12, GLP-1RA users required lower point-in-time ESA doses (29.1 vs 31.3 μg; mean difference −2.2, 95% CI, −3.87 to −0.53) (Fig 3B, Table 3) and had lower cumulative 12-month ESA exposure (1,881.9 vs 2,005.8 μg; mean difference −123.9 μg, 95% CI, −201.7 to −46.1) (Fig 3C, Table 3). ERI, a composite measure of ESA responsiveness, improved (decreased) in the treatment group, with separation occurring by month 3 (3.26 vs 3.60) and sustained through month 12 (3.31 vs 3.63; mean difference −0.32, 95% CI, −0.54 to −0.10) (Fig 3D, Table 3).

These changes were accompanied by indicators of higher iron availability. Transferrin saturation value increased over time, reaching 34.0% in the treatment group compared with 32.3% in controls at month 12 (mean difference 1.7, 95% CI, 0.98-2.42) (Fig 2A, Table 3). Ferritin followed a similar upward trajectory, remaining higher in the GLP-1RA group through month 12 (887.4 vs 846.9 ng/mL) (Fig 2B, Table 3). Serum iron levels showed a modest increase over time in the GLP-1RA group compared with controls (Fig 2C).

Although the GLP-1RA group exhibited higher hemoglobin levels at months 2 and 3, levels were similar by month 12 (10.90 vs 10.84 g/dL) (Fig 3A, Table 3). The average IV iron dose per administration was lower in the GLP-1RA group at month 12 (74.2 vs 76.4 mg; mean difference −2.2, 95% CI, −3.79 to −0.61), suggesting a reduced requirement for exogenous iron supplementation (Fig 4A, Table 3).

### Sensitivity Analyses

In sensitivity analyses incorporating time-varying adjustment for body weight and IV iron dosing, the observed trajectories and between-group differences remained materially unchanged across all outcomes (Figs S1-S11). These findings suggest that the associations observed in the primary analyses are robust and not materially influenced by differences in weight change or iron supplementation over time.

## Discussion

In this large, retrospective study of patients receiving HD over 12 months, GLP-1RA users demonstrated sustained reductions in NLR and WBC counts compared with nonusers. These anti-inflammatory effects were accompanied by biomarker evidence of higher iron-related parameters (including ferritin and transferrin saturation), reduced ESA utilization, and lower ERI, while maintaining similar hemoglobin levels. Although the serum ferritin level is traditionally used as an indicator of iron stores, it is also a well-established acute-phase reactant in the hemodialysis population. In our study, changes in ferritin occurred alongside directional changes in other markers associated with systemic inflammation, including NLR and WBC counts. However, given the absence of established inflammatory biomarkers such as high-sensitivity C-reactive protein (hs-CRP) levels, we cannot distinguish whether these trajectories primarily reflect alterations in iron metabolism, inflammatory status, or a combination of both. Given the number of related outcomes evaluated, these analyses should be interpreted as hypothesis-generating. Emphasis should be placed on the consistency of directional changes across parameters rather than any individual endpoint. Collectively, these findings suggest that GLP-1RA use is associated with a reduction in inflammatory markers and modest improvements in iron utilization and ESA-related parameters in the population of patients with KFRT receiving HD. These observations raise the possibility that the pleiotropic anti-inflammatory effects of GLP-1RAs may contribute to attenuation of “anemia of inflammation,” although causal inference cannot be established.

Considerable attention has been justifiably paid to the effect of the GLP-1RA on reducing major adverse cardiovascular events (MACE) in a variety of patient populations. The Evaluate Renal Function with Semaglutide Once Weekly (FLOW) trial^6^ and the Semaglutide Effects on Heart Disease and Stroke in Patients With Overweight or Obesity (SELECT) trial^7^ demonstrated a significant reduction in MACE in patients with CKD prescribed semaglutide compared with placebo.^4^ To date, there has been no prospective, randomized, controlled trial of a GLP-1RA on MACE (or any other clinical outcome) in the KFRT population. However, a recent retrospective analysis using the United States Renal Data System data reported a 23% rate reduction in mortality among patients on hemodialysis using any GLP-1RA compared with matched controls (hazard ratio, 0.77; 95% CI, 0.70-0.85; *P* < .001).^8^

Anemia is a common complication of advanced CKD and KFRT. The etiology of anemia of renal disease is multifactorial and often includes a reduction of iron stores or a limitation in its availability and a deficiency of erythropoietin. All these mechanisms may be caused or exacerbated by inflammation, and the salutary effect of the GLP-1RA on MACE outcomes in CKD (and perhaps in end-stage kidney disease) may be partially explained by an attenuated systemic inflammatory milieu. Elevated circulating hs-CRP levels have been associated with both increased rates of MACE^4^ as well as anemia of inflammation.^4^ Systemic inflammation associated with kidney disease has been associated with hepcidin-mediated defects in iron utilization as well as ESA resistance,^9^ a phenomenon known as “anemia of inflammation.” Elevated hs-CRP levels have also been associated with systemic inflammation and ESA resistance.^10^ Targeted therapies to reduce systemic inflammation using interleukin-6 receptor antagonists have been shown in small studies to significantly reduce circulating hs-CRP levels.^11^^,^^12^ Early studies using the interleukin-6 receptor antagonist ziltivekimab suggest improved anemia and iron parameters while also reducing iron utilization in both patients with KFRT^6^^,^^12^ and CKD stages 3-5.^6^ Two renal-focused phase 3 studies of interleukin-6 receptor antagonists with MACE primary endpoints are currently underway: The Research Study to Look at How Ziltivekimab Works Compared with Placebo in People With Cardiovascular Disease, Chronic Kidney Disease and Inflammation (ZEUS) trial is examining the effect of ziltivekimab on MACE in an advanced CKD population^13^ and the Combined Dose-Finding and CV Outcomes Study With CSL300 (Clazakizumab) in Adult Subjects With ESKD Undergoing Dialysis (POSIBIL6ESKD) trial is examining the effect of clazakizumab on MACE in a KFRT population.^14^ The ZEUS and POSIBIL6ESKD trials will both include anemia laboratory parameters and ESA utilization as secondary study endpoints, predicated on the premise that reductions in hs-CRP levels are associated with improvements in anemia because of inflammation.

GLP-1RA use has also been shown to reduce hs-CRP levels. The Effect of Semaglutide 2.4 mg Once Weekly on Function and Symptoms in Subjects With Obesity-related Heart Failure With Preserved Ejection Fraction (STEP-HFpEF) trial, which studied the impact of weekly 2.4 mg semaglutide in patients with heart failure with preserved ejection fraction and obesity, showed a significant reduction in hs-CRP level compared with placebo.^15^ An analysis of the STEP 1, 2, and 3 trials showed that lower (1.0 mg) doses of semaglutide resulted in significant reductions in hs-CRP levels, a finding independent of significant weight loss.^3^ A meta-analysis of 13 randomized controlled trials of semaglutide showed a greater effect of hs-CRP level reduction in patients without type 2 diabetes compared with those with type 2 diabetes,^2^ though the rates of reduction of hs-CRP levels were not segmented by CKD stage or status. The FLOW trial,^7^ which did include patients with advanced CKD, did not measure hs-CRP in the study enrollees. hs-CRP levels are not routinely measured in US patients with KFRT, but our analysis also included an assessment of the NLR value in the GLP-1 and non-GLP-1 groups. An elevated NLR value has been correlated in some studies with elevated hs-CRP levels,^16^ and an elevated NLR value has also been associated with erythropoietin resistance.^17^ Our study showed that NLR value divergence began early (month 2) and persisted through month 12 (see Table 2). This trend at least raises the possibility that the observed association of GLP-1RA on anemia parameters, point-in-time and cumulative ESA use, and ERI may be related to attenuation of systemic inflammation.^13^^,^^14^ However, the absolute magnitude of change in several laboratory parameters was modest, and the clinical significance of these differences remains uncertain. Sensitivity analyses incorporating time-varying adjustment for body weight and IV iron dosing yielded similar results (see Results), supporting the robustness of the findings.

Our study has several limitations. This is a retrospective study using propensity-matched scoring to identify a control arm and is therefore subject to residual confounding. The requirement that patients have 12 months of follow-up data and the exclusion of patients who died or received a transplant during the study may have introduced survivorship bias. Home medication data are not always reliably collected in the outpatient dialysis clinic setting, although we do not expect any bias to result from this issue. As pre-KFRT type, dose, indication, and duration of use of the GLP-1RA were not available, discerning differences in efficacy of 1 type, dose, and/or duration of use of a particular GLP-1RA was not possible. Our data set was not designed to assess medication adherence over the course of the study. Some laboratory variables included in the model may lie on the causal pathway between inflammation and ESA use. Their inclusion may reduce residual confounding but may also attenuate true associations. hs-CRP level is not routinely measured in the US dialysis population, and so an association between hs-CRP levels, GLP-1RA use, and the laboratory and anemia-related medication utilization rates under study was not possible. Further, complete blood count with differential is not always measured monthly, potentially resulting in fluctuations when looking at monthly averages. While the cohort was predominantly treated with semaglutide, we did not account for potential differences in anti-inflammatory potency between specific GLP-1RA agents or dose-dependent effects. Future studies should investigate whether newer-generation agents like tirzepatide offer superior pleiotropic benefits compared with older GLP-1RA therapies. Finally, as an observational matched cohort study, residual confounding cannot be excluded despite adjustment for measured baseline characteristics. In particular, unmeasured factors such as health-seeking behavior, provider prescribing patterns, or socioeconomic differences may contribute to treatment selection, raising the possibility of healthy-user or treatment selection bias. As such, GLP-1RA users may represent a comparatively healthier population, and this potential “healthy user” or treatment selection bias cannot be fully accounted for in this observational analysis.

Although use of other glucose-lowering therapies, including dipeptidyl peptidase-4inhibitors, was balanced between groups, the absence of an active comparator design (eg, comparison with another glucose-lowering agent) limits causal interpretation. As such, the observed differences may partially reflect underlying differences between patients selected for GLP-1RA therapy versus those not receiving these agents.

A randomized, double-blinded, standard-of-care placebo-controlled trial of the effect of a GLP-1RA on MACE outcomes in the KFRT population is unlikely to be performed because of the combined challenges of recruiting a significant number of patients to reach appropriate statistical power and following recruited patients over several years to observe a sufficient number of MACE events in both study arms. However, this retrospective study does offer some support to a hypothesis that an appropriately powered randomized, controlled trial of a GLP-1RA in the KFRT population focused on anemia and iron laboratory parameters, reduced circulating hs-CRP levels, anemia medication utilization, and key shorter-term safety and clinical endpoints may be both feasible and clinically warranted.

In summary, GLP-1RA use in patients receiving hemodialysis was associated with small but consistent changes over 12 months, including reductions in inflammatory markers (NLR value and WBC count) and improvements in nutritional status (albumin), improved iron parameters, noninferior anemia parameters, lower cumulative ESA requirements, and reduced ESA resistance. This analysis suggests that GLP-1RA use in KFRT may be associated with improvements in markers related to inflammation and anemia beyond glycemic and weight control. As an exploratory, real-world analysis, these findings should be interpreted as signals of potential improvement rather than definitive clinical outcomes. Although the shift in biomarkers like NLR values and albumin levels is statistically robust, the absolute magnitude of these changes is modest, and their long-term impact on clinical endpoints requires further investigation.

## Supplemental Material

Supplementary File (PDF) Figured S1-S11; Table S1.
