# Supplementary material for: Real-world Evidence for Improvements in Inflammation and Anemia Biomarkers After the Initiation of GLP-1RA in Patients Receiving Hemodialysis
Source: Kidney Med. 2026 Jul 17;8(10):101476. doi: 10.1016/j.xkme.2026.101476 (PMC13602310; doi:10.1016/j.xkme.2026.101476)
Supplement: Supplementary file 1 [file mmc1.pdf]

Table S1: Baseline characteristics of unmatched GLP-1RA users compared to the eligible control pool.

	Mean ± SD or %		
	**Non-GLP1 User**	**GLP-1RA: Not Selected**	**Standardized Difference**
Patient n	73328	215	
**Demography**			
Age (years)	63.9 ± 13.9	55.96 ±12.39	-0.603
Dialysis vintage (years)[1]	4.89 ± 4.4	1.75 ±2.33	-0.895
Female	30769 (42.0%)	114 (53.0%)	0.213
White Race	37046 (50.5%)	100 (46.5%)	-0.080
Black Race	28352 (38.7%)	81 (37.6%)	-0.011
Other Race	6159 (8.4%)	21 (9.8%)	0.048
Unknown Race	1771 (2.4%)	13 (6.0%)	0.162
BMI	28.4 ± 7.1	42.37 ±10.48	1.563
**Lab Parameters**			
TSAT (%)	33.56 ± 15.68	26.61 ±11.89	-0.493
Albumin (g/dL)	3.92 ± 0.35	3.86 ±0.36	-0.169
NLR	4.09 ± 3.15	4.29 ±2.36	0.085
Hemoglobin (g/dL)	10.75 ± 1.27	10.48 ±1.48	-0.195
Iron (mcg/dL)	75.3 ± 34.92	63.98 ±24.92	-0.370
Ferritin (ng/mL)	906.53 ± 488.82	551.29 ±455.44	-0.736
WBC (1000/mcL)	6.7 ± 2.45	8.07 ±2.53	0.533
**Medication**			
ESA (mcg)	27.00 ± 30.6	9.7 ±17.41	-0.693
IV Iron (mg)	54.3 ± 37.4	27.44 ±36.37	-0.729
**Insurance**			
Commercial Insurance	5,647 (7.7%)	43 (20.0%)	0.362
Medicaid Insurance	7,197 (9.8%)	22 (10.2%)	0.0139
Medicare Insurance	57,707 (78.7%)	144 (67.0%)	-0.256
Others†	2,777 (3.8%)	6 (2.8%)	0.06
**Comorbidities**			
Diabetes	46239 (63.1%)	135 (62.7%)	0.004
Coronary artery disease	18,955 (25.8%)	56 (26.0%)	-0.315
Peripheral Artery Disease	5,882 (8.0%)	3 (1.4%)	-0.317
Congestive heart failure	18,555 (25.3%)	45 (20.9%)	-0.115
Cerebrovascular Disease	5,482 (7.5%)	1 (0.5%)	-0.365
Myocardial Infarction	12,699 (17.3%)	22 (10.2%)	-0.207
History of Malignancy	3,014 (4.1%)	2 (0.9%)	-0.204
ERI	2.7 ± 1.8	1.9 ±1.3	-0.527

Table S1: The unmatched cohort (n=215) exhibited large standardized differences (absolute standardized difference >0.2) compared to the control pool

1 Dialysis vintage was defined as the time from first dialysis treatment to the GLP-1RA initiation (index) date.

† 'Others' insurance types included insurance like Veterans Affairs, Self-pay, and Exchange plans etc.

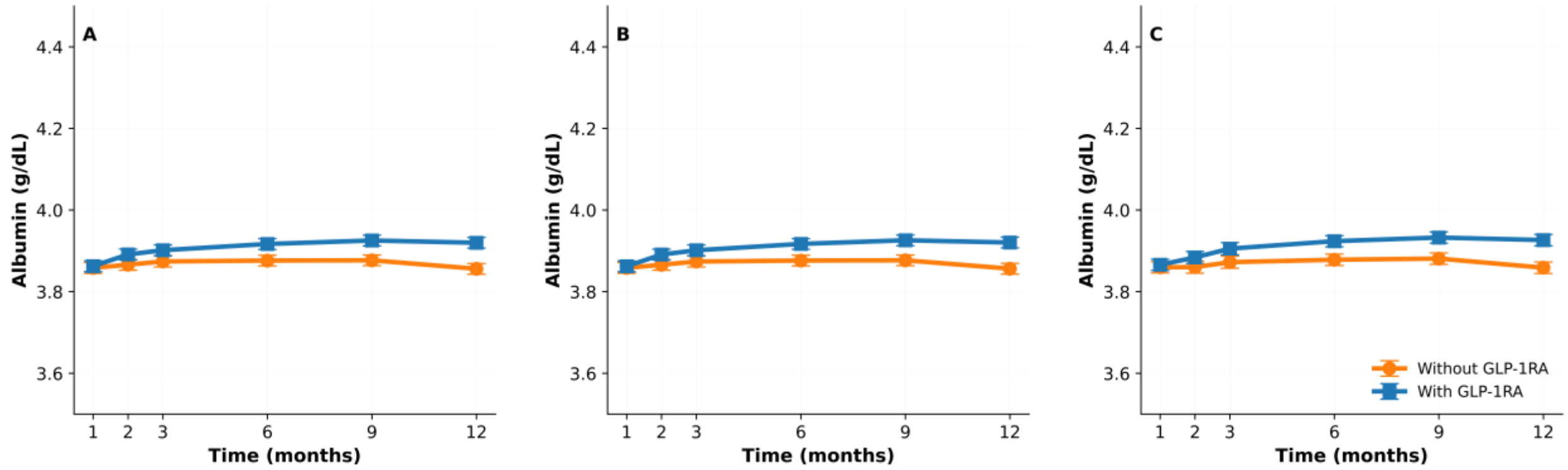

Figure S1. Longitudinal albumin trends in GLP-1RA users and matched controls under unadjusted (A), weight-adjusted (B), and IV iron–adjusted (C) models. Trajectories remained consistent across models, supporting the robustness of the primary analysis.

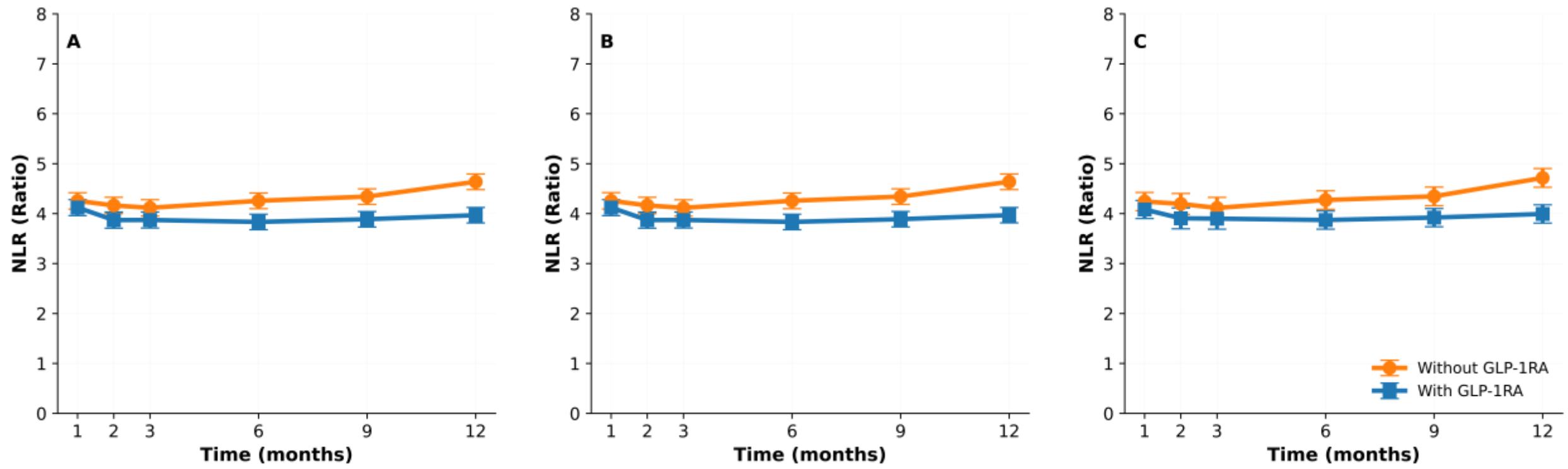

Figure S2. Longitudinal neutrophil-to-lymphocyte ratio (NLR) trends in GLP-1RA users and matched controls under unadjusted (A), weight-adjusted (B), and IV iron–adjusted (C) models.

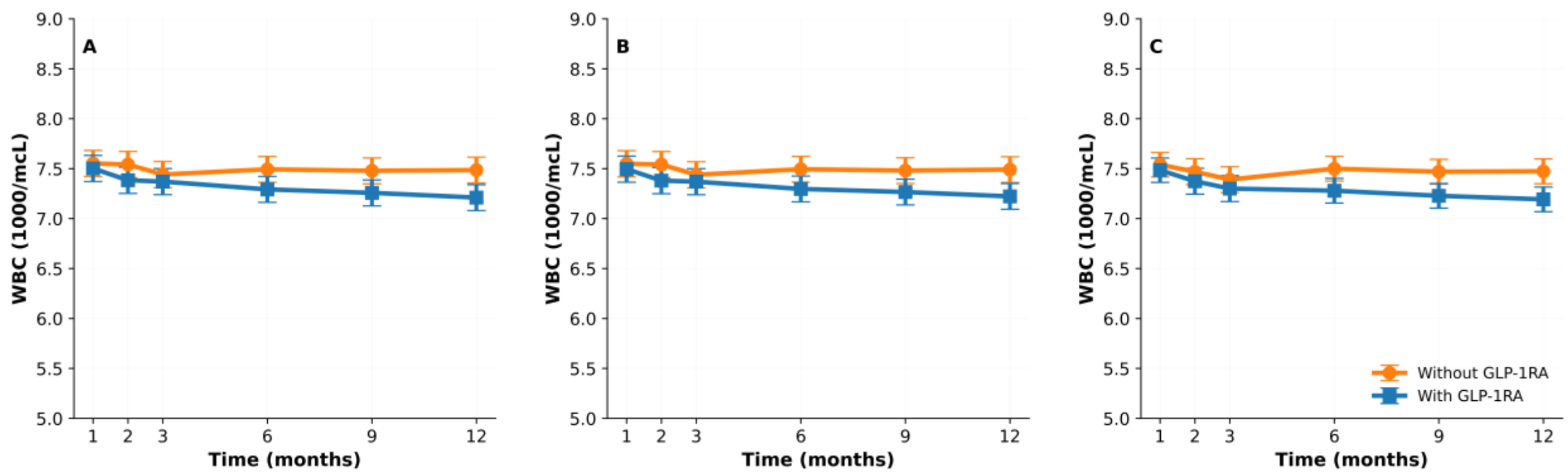

Figure S3. Longitudinal white blood cell (WBC) trends in GLP-1RA users and matched controls under unadjusted (A), weight-adjusted (B), and IV iron–adjusted (C) models.

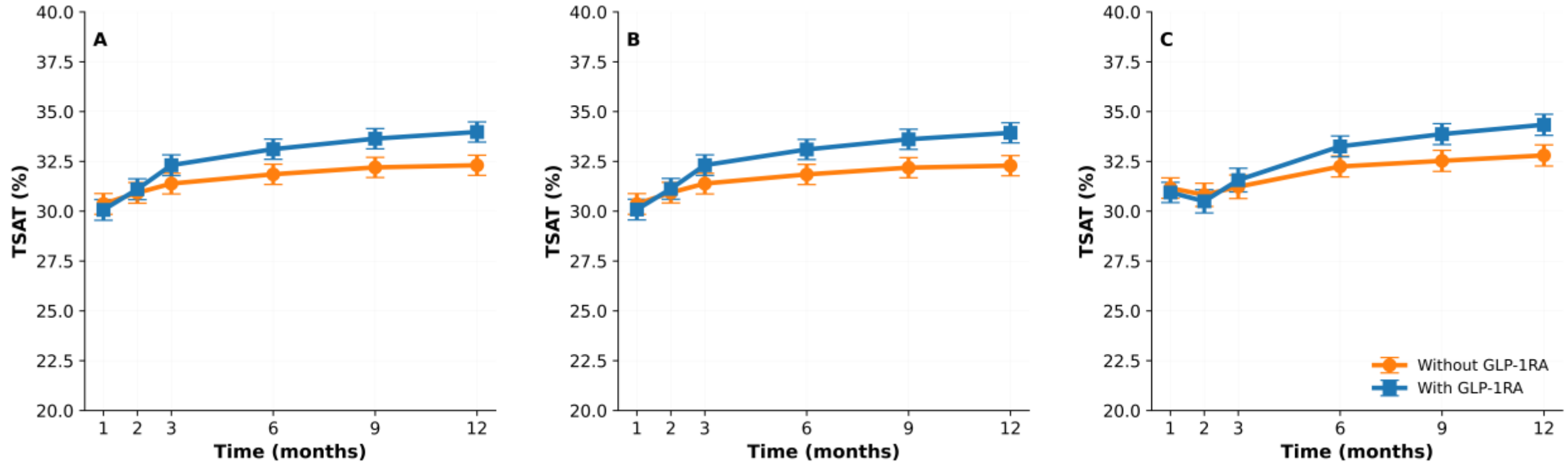

Figure S4. Longitudinal transferrin saturation (TSAT) trends in GLP-1RA users and matched controls under unadjusted (A), weight-adjusted (B), and IV iron–adjusted (C) models.

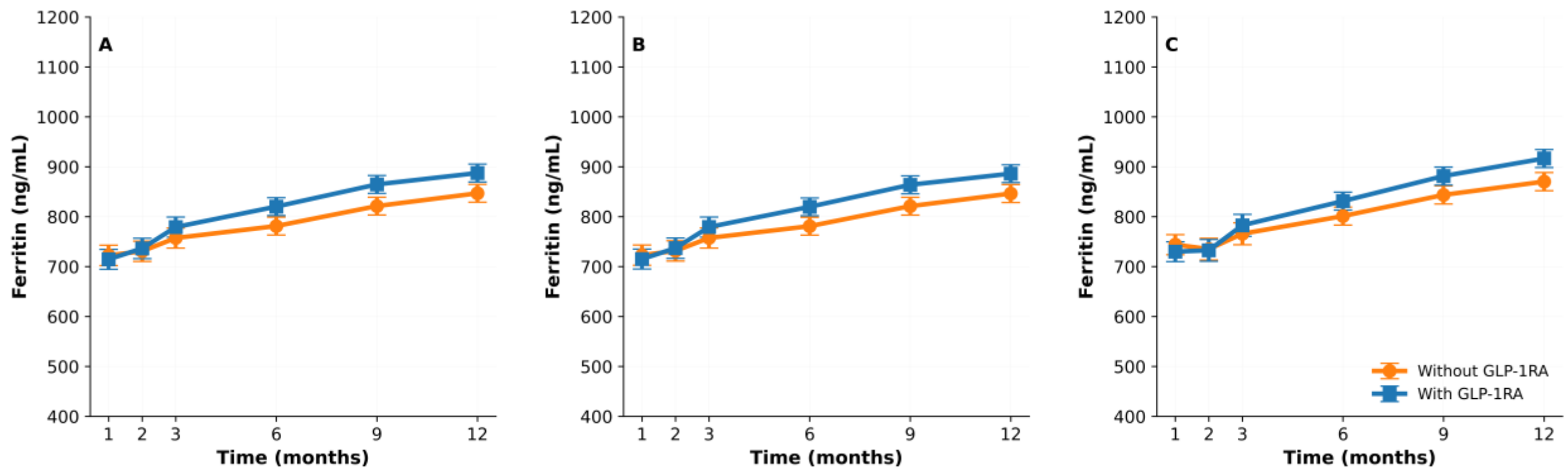

Figure S5. Longitudinal ferritin trends in GLP-1RA users and matched controls under unadjusted (A), weight-adjusted (B), and IV iron–adjusted (C) models.

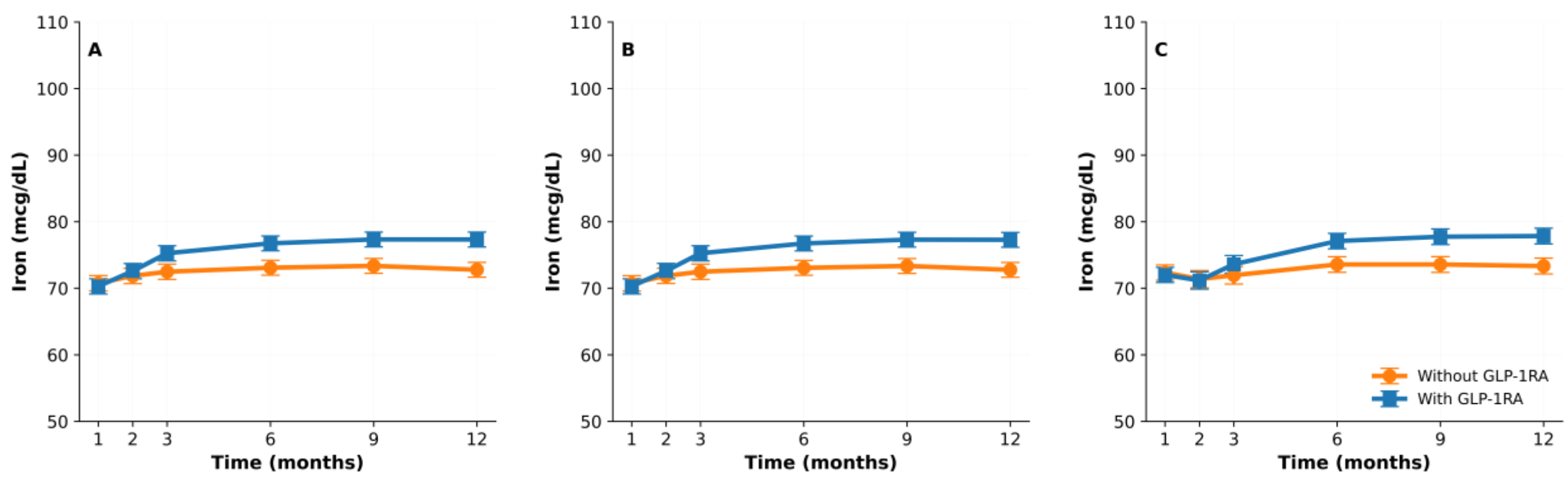

Figure S6. Longitudinal serum iron trends in GLP-1RA users and matched controls under unadjusted (A), weight-adjusted (B), and IV iron–adjusted (C) models.

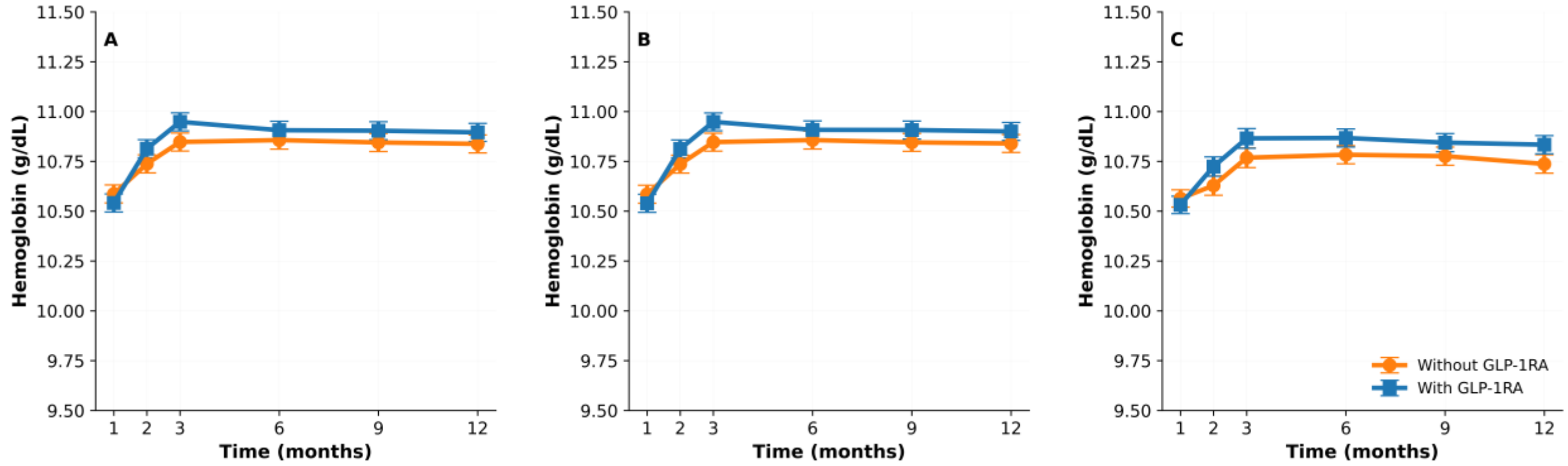

Figure S7. Longitudinal hemoglobin trends in GLP-1RA users and matched controls under unadjusted (A), weight-adjusted (B), and IV iron–adjusted (C) models.

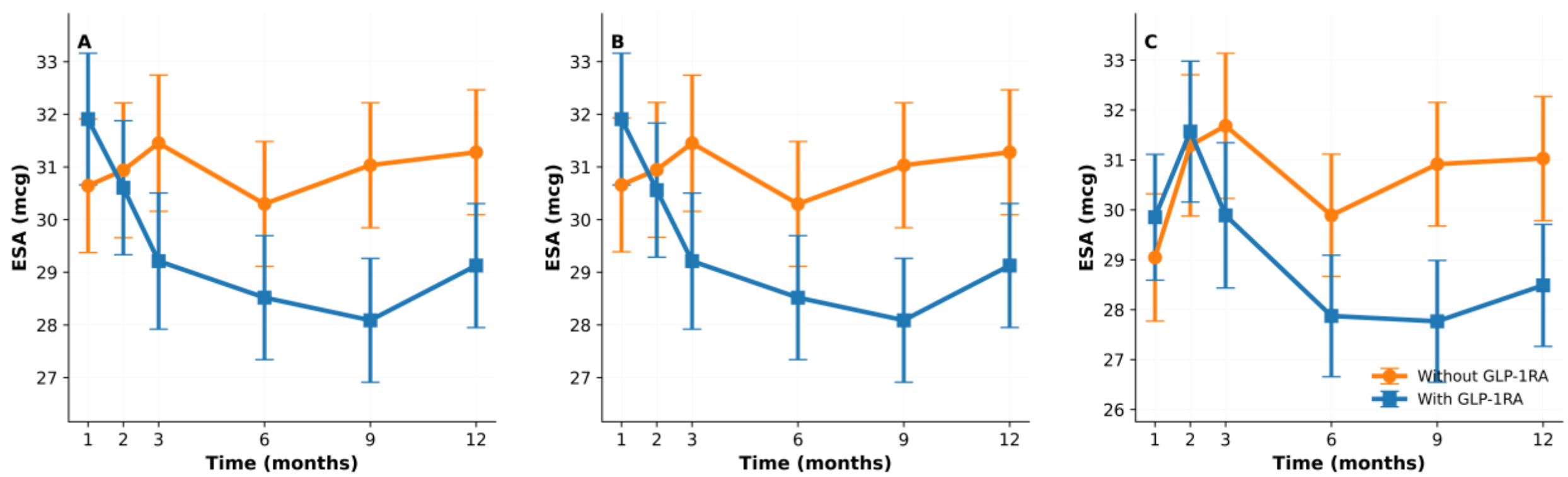

Figure S8. Longitudinal erythropoiesis-stimulating agent (ESA) dose trends in GLP-1RA users and matched controls under unadjusted (A), weight-adjusted (B), and IV iron–adjusted (C) models.

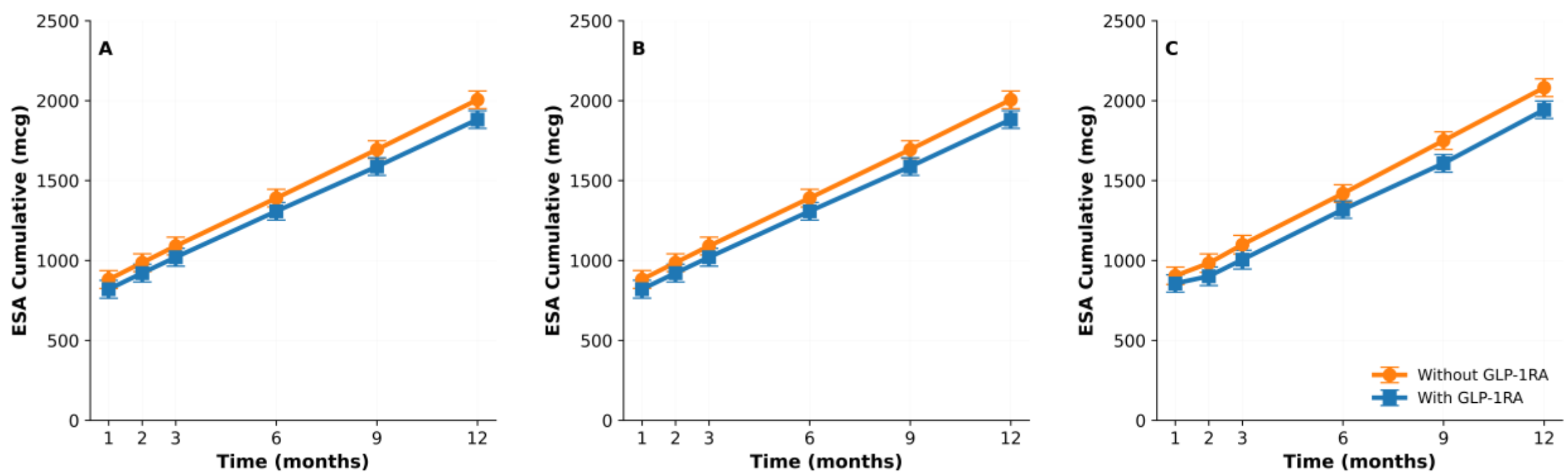

Figure S9. Longitudinal cumulative ESA exposure trends in GLP-1RA users and matched controls under unadjusted (A), weight-adjusted (B), and IV iron–adjusted (C) models.

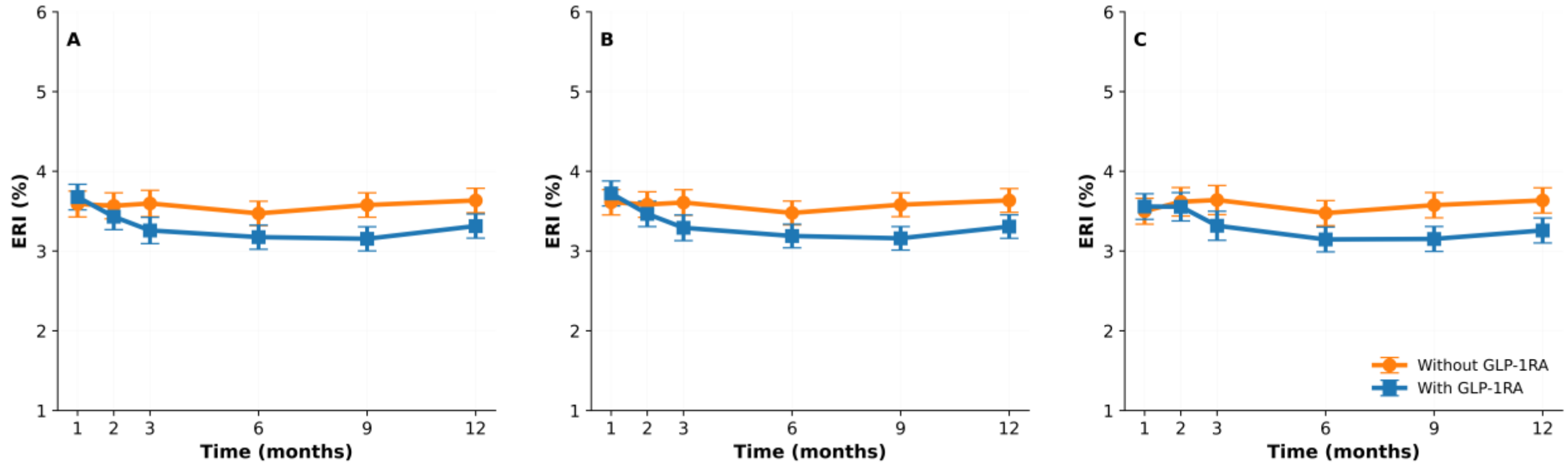

Figure S10. Longitudinal erythropoietin resistance index (ERI) trends in GLP-1RA users and matched controls under unadjusted (A), weight-adjusted (B), and IV iron–adjusted (C) models.

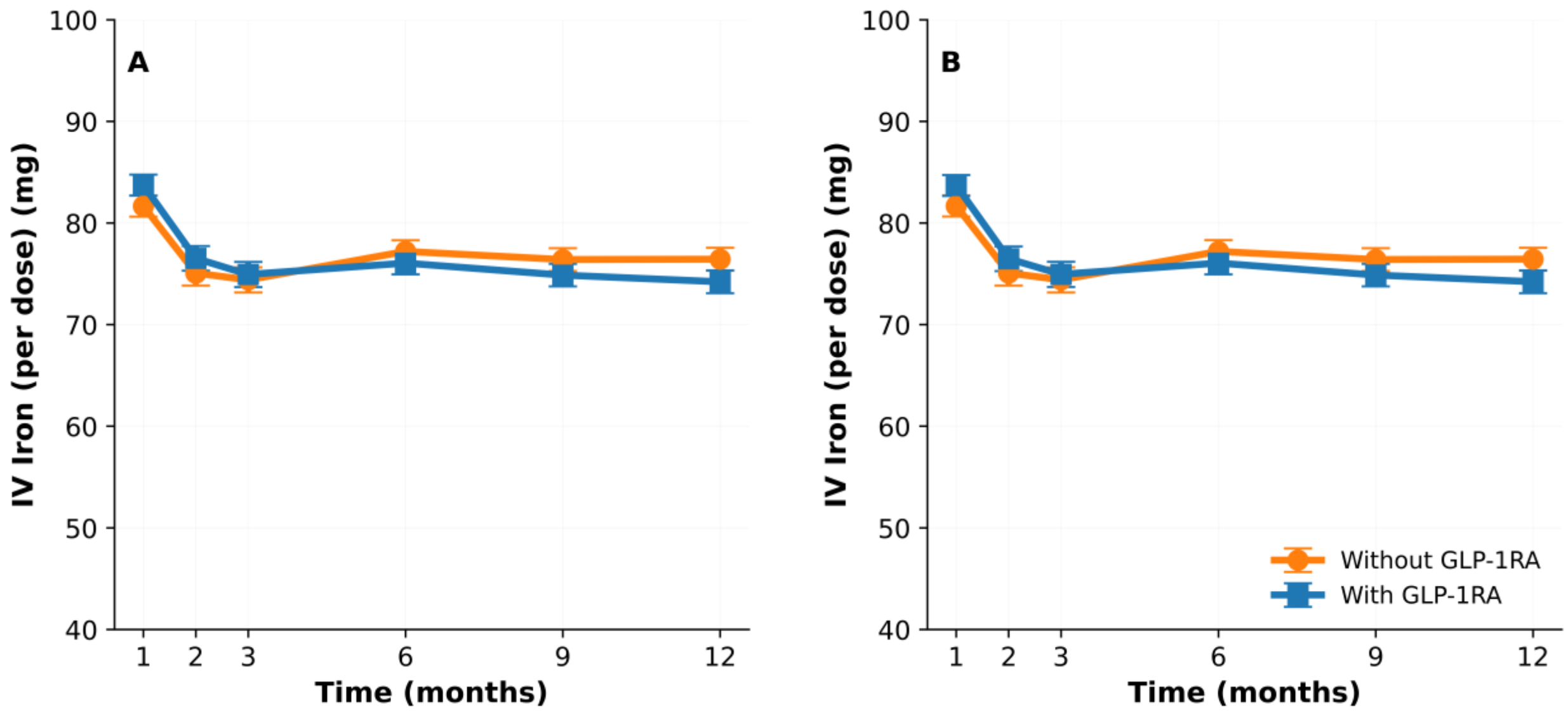

Figure S11. Longitudinal intravenous (IV) iron dose trends in GLP-1RA users and matched controls under unadjusted (A) and weight-adjusted (B) models.
